# *QuickStats*: Age-Adjusted Percentage of Adults Aged ≥20 Years Who Tried to Lose Weight During the Past 12 Months,* by Sex — National Health and Nutrition Examination Survey, 2007–2008 to 2015–2016

**DOI:** 10.15585/mmwr.mm6741a10

**Published:** 2018-10-19

**Figure:**
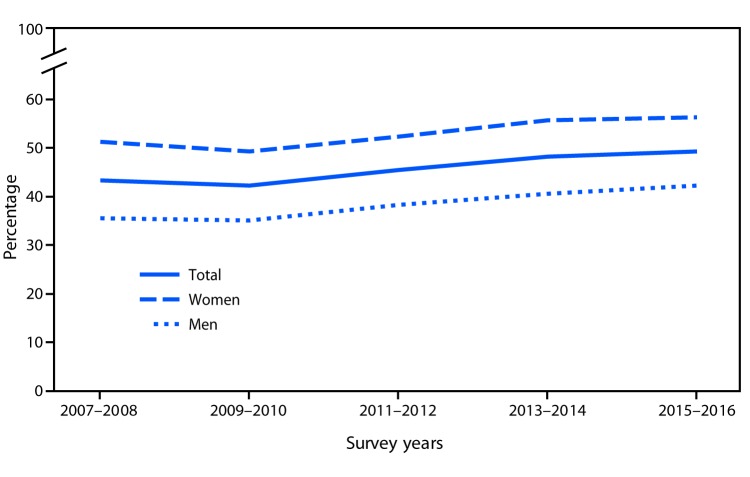
From 2007–2008 to 2015–2016, the age-adjusted percentage of adults who tried to lose weight during the past 12 months increased from 43.3% to 49.3%. This increase was seen among both men (35.5% to 42.2%) and women (51.2% to 56.3%). The percentage of women who tried to lose weight in the past year was higher than that for men for each survey year from 2007–2008 to 2015–2016.

**Source:** National Center for Health Statistics data brief no. 313. https://www.cdc.gov/nchs/data/databriefs/db313.pdf; National Center for Health Statistics, National Health and Nutrition Examination Survey Data, 2007–2008 to 2015–2016. https://www.cdc.gov/nchs/nhanes.htm.

